# 1α,25-Dihydroxyvitamin D_3_ Ameliorates Seawater Aspiration-Induced Acute Lung Injury via NF-κB and RhoA/Rho Kinase Pathways

**DOI:** 10.1371/journal.pone.0104507

**Published:** 2014-08-13

**Authors:** Minlong Zhang, Mingqing Dong, Wei Liu, Li Wang, Ying Luo, Zhichao Li, Faguang Jin

**Affiliations:** 1 Department of Respiration, Tangdu Hospital, Fourth Military Medical University, Xi'an, PR China; 2 Department of Pathology and Pathophysiology, Fourth Military Medical University, Xi'an, PR China; 3 Lung Injury and Repair Center, Fourth Military Medical University, Xi'an, PR China; Helmholtz Zentrum München, Germany

## Abstract

**Introduction:**

Inflammation and pulmonary edema are involved in the pathogenesis of seawater aspiration-induced acute lung injury (ALI). Although several studies have reported that 1α,25-Dihydroxyvitamin D_3_ (calcitriol) suppresses inflammation, it has not been confirmed to be effective in seawater aspiration-induced ALI. Thus, we investigated the effect of calcitriol on seawater aspiration-induced ALI and explored the probable mechanism.

**Methods:**

Male SD rats receiving different doses of calcitriol or not, underwent seawater instillation. Then lung samples were collected at 4 h for analysis. In addition, A549 cells and rat pulmonary microvascular endothelial cells (RPMVECs) were cultured with calcitriol or not and then stimulated with 25% seawater for 40 min. After these treatments, cells samples were collected for analysis.

**Results:**

Results from real-time PCR showed that seawater stimulation up-regulated the expression of vitamin D receptor in lung tissues, A549 cells and RPMVECs. Seawater stimulation also activates NF-κB and RhoA/Rho kinase pathways. However, we found that pretreatment with calcitriol significantly inhibited the activation of NF-κB and RhoA/Rho kinase pathways. Meanwhile, treatment of calcitriol also improved lung histopathologic changes, reduced inflammation, lung edema and vascular leakage.

**Conclusions:**

These results demonstrated that NF-κB and RhoA/Rho kinase pathways are critical in the development of lung inflammation and pulmonary edema and that treatment with calcitriol could ameliorate seawater aspiration-induced ALI, which was probably through the inhibition of NF-κB and RhoA/Rho kinase pathways.

## Introduction

Drowning is one of the most common causes of death and remains serious social and medical issue. It is estimated that drowning results in more than one half million deaths each year and seawater drowning account for a large part [1]. Despite decades of intensive research, the mortality rate remains high. The most significant pathophysiological change in seawater drowning is hypoxia [2]. Seawater aspiration can also bring about acute lung injury (ALI) or acute respiratory distress syndrome (ARDS). The seawater aspiration-induced ALI/ARDS that develops due to the effects of surfactant disruption, alveolar collapse, atelectasis and intrapulmonary shunting is characterized by up-regulation of inflammatory mediators, severe dyspnea, serious hypoxemia and edema [3]. In seawater aspiration-induced ALI animal models, the inflammatory cells which have infiltrated into alveolar spaces release several molecules, such as pro-inflammatory cytokines, reactive oxygen species, contributing to the pulmonary inflammatory process. In addition, lung edema is prominent in seawater induced ALI because high osmotic seawater pull water through vascular endothelial cells and alveolar epithelial cells [4], [5]. It has been reported that increased alveolar epithelial and pulmonary microvascular endothelial permeability trigger the transmigration of inflammatory cells such as neutrophils and the formation of edema fluid [6]. Accordingly, inhibition of inflammation and lung tissue barrier permeability may be propitious to the promotion of seawater aspiration-induced ALI.

One of the most widely recognized intracellular signaling pathway in inflammatory responses is the nuclear factor kappa B (NF-κB) signaling pathway. NF-κB is expressed in almost all cells [7], and regulates various genes involved in immune and acute phase inflammatory reaction [8]. Many pro-inflammatory stimulus can cause the activation of NF-κB through the phosphorylation of inhibitors of κB (IκBs) by the IκB kinase (IKK) complex [9]. Afterwards, the freed NF-κB translocate into the nucleus in which it can lead to the transcriptional activation of several pro-inflammatory mediators, including TNF-α, IL-1β and IL-6, via recognizing κB binding sites on their target genes [10].

Rho and its target protein, Rho-associated coiled-coil forming protein kinase (ROCK) pathways a calcium-sensitizing signaling pathway implicated in the cytoskeletal contractile response through their influence on myosin ATPase activity [11]. ROCK causes phosphorylation of the light-chain of myosin (MLC) on Ser 19 and Thr 18 via phosphorylation of the MLC phosphatase and suppression of MLC dephosphorylation [12]. The phosphorylation of MLC ultimately promotes myosin ATPase activity, resulting in the actin myosin reorganization, tension fiber formation and cell contraction [12]. Therefore, Rho and ROCK are well-established mediators of the permeability between cells. In addition, it has been shown that RhoA/ROCK pathway plays an important role in regulation of the inflammation response [13], [14]. Although the RhoA/ROCK pathway has been confirmed at several mechanistic levels [15], the real role of this pathway in ALI induced by seawater aspiration is still unknown.

1α,25-Dihydroxyvitamin D_3_ (calcitriol), the active form of vitamin D, has traditionally associated with cellular differentiation and proliferation, calcium and phosphorus homeostasis, bone mineralization, and immunomodulation. Calcitriol achieves these physiological roles by binding to its nuclear receptor, the vitamin D receptor (VDR) [16]. Recently, calcitriol was found to be related with inflammation response. It has been observed that calcitriol can inhibit neutrophil recruitment and pro-inflammatory cytokines release in lipopolysaccharide (LPS)-induced ALI [17]. Besides, vitamin D receptor agonist can significantly reduce the actin-dependent cytoskeletal rearrangement through RhoA/ROCK pathway in the benign prostatic hyperplasia cells [18]. However, whether calcitriol can inhibit inflammation and lung epithelial-endothelial barrier permeability in seawater aspiration-induced ALI remains unclear. Corticoids is a drug option in the clinical therapy of ALI/ARDS and dexamethasone treatment has been proved to have therapeutic effects in seawater aspiration-induced ALI [4]. Therefore, we choose dexamethasone as a positive control drug.

In our study, we firstly investigated that the effect of calcitriol pretreatment ameliorates seawater aspiration-induced ALI via inhibition of inflammatory responses and alleviation of lung epithelial-endothelial barrier permeability. In addition, we also demonstrated that the probable mechanism of these effects was through inhibition of NF-κB and RhoA/ROCK pathways activation. This finding proved that calcitriol might be a potential agent in the prevention and therapy of the seawater aspiration-induced ALI.

## Methods

### Animals and experimental protocol

Male SD rats (5–7 weeks old) weighing 200±20 g were obtained from the Animal Center of Fourth Military Medical University. The rats were kept in a temperature-controlled house with 12 h light-dark cycles and free access to standard laboratory diet and water ad libitum. All the animal experiments were approved by the Animal Care and Use Committee of the Fourth Military Medical University and in accordance with the Declaration of the National Institutes of Health Guide for Care and Use of Laboratory Animals (Publication No. 85-23, revised 1985).

### Drug and reagents

Calcitriol (HPLC purity≥99%) and dexamethasone (HPLC purity≥98%) were purchased from Sigma-Aldrich Company (St. Louis, MO, USA). Seawater (osmolality 1300 mmol/L, PH 8.2, SW 1.05, NaCl 6.518 g/L, MgSO_4_ 3.305 g/L, MgCl_2_ 2.447 g/L, CaCl_2_ 1.141 g/L, KCl 0.725 g/L, NaHCO_3_ 0.202 g/L, NaBr 0.083 g/L) was prepared according to the major composition of the East China Sea provided by Chinese Ocean Bureau. Enzyme-linked immunosorbent assay (ELISA) kits for TNF-α, IL-1β and IL-6 were obtained from R&D Systems (Minneapolis, MN, USA). Anti-RhoA, anti-MYPT1, anti-pMYPT1, anti-CD31 and anti-β-actin antibodies were purchased from Santa Cruz Biotechnology Inc. (Santa Cruz, CA, USA). Anti-NF-κB p65, anti-pNF-κB p65 antibodies were purchased from Cell Signaling Technology (Cell Signaling, MA, USA).

### Model and grouping

Seawater Group (SG): The seawater aspiration model was produced according to the method described in previous reports with some modifications. The rats were anesthetized with pentobarbital sodium (100 mg/kg) intraperitoneally. The rats were maintained in the supine position during experiments with the head elevated 30°. A heparin-filled blunt-ended polyethylene catheter was inserted into the left carotid artery to monitor the mean arterial pressure and obtain blood samples. After exposure of the trachea, a 20 min stable baseline period was followed, and then a 1 cm syringe was gently inserted into the trachea approximately 1.5 cm above the carina. Next, seawater (4 ml/kg) was instilled at a steady speed within 4 min into both lungs.

Calcitriol Group (CG): Calcitriol was dissolved in vehicle (10% (v/v) ethanol-0.1% (v/v) Triton X-100 in saline). 1 µg/kg, 5 µg/kg and 25 µg/kg calcitriol was respectively injected perorally twice at 48 h and 24 h before seawater instillation, and the other treatment conditions were the same as the SG's.

Dexamethasone Group (DG): Dexamethasone was dissolved in vehicle (0.5% (w/v) sodium carboxymethylcellulose in distilled water). Rats were administered dexamethasone perorally (10 mg/kg) twice at 48 h and 24 h before seawater instillation, and the other treatment conditions were the same as the SG's.

Normal Group (NG): Seawater, calcitriol and dexamethasone were not given in this group, and the other treatment conditions were the same as the SG's.

At the end of the experiments, the rats were exsanguinated by aortic transaction at 4 h after seawater instillation. The thorax was opened rapidly and lungs were processed in the manners described below.

### Lung morphology

For lung histological studies, the rats were killed at 4 h after seawater exposure. The same right lower lung lobes from every rat were preserved in 10% formalin for 24 h, and then embedded in paraffin wax, sliced and stained with hematoxylin-eosin. Microscopic evaluation was performed to characterize lung injury.

### Lung wet/dry weight ratio

Lung wet/dry (W/D) ratio was used to represent the severity of pulmonary edema. The left lung lobes were obtained 4 h after seawater instillation and weighed immediately after removal, and then dried to constant weight at 50°C for 72 h and weighed again. The W/D ratio was calculated by dividing the wet weight by the dry weight.

### Assessment of extravasation of Evans blue

Pulmonary barrier permeability was assessed by the Evans blue dye extravasation. Evans blue dye (20 mg/kg) was injected to the rats 30 min before anesthesia. At the end of the experiment, normal saline was immediately injected into the right ventricle till effused clear fluid from the left atrium. The right middle lung lobe was removed and dried to a constant weight at 60°C for 72 h. Evans blue dye was extracted from the lung lobes by incubation at room temperature for 24 h in formamide (3 ml/100 mg). The optical density of the supernatant was determined spectrophotometrically at 620 nm. The total amount of Evans blue (µg/g tissue) was calculated against the generated Evans blue standard absorbance curves.

### Measurement of pro-inflammation cytokines

The levels of TNF-α, IL-1β and IL-6 were measured with TNF-α, IL-1β and IL-6 ELISA kits according to the manufacturer's protocol. Briefly, portions of lung tissues were homogenized in cool phosphate-buffered saline (lung tissue to normal saline 1∶5). When the tissue lysate was collected, the concentration of total protein in tissue lysate was detected and then all samples were made to the same concentration by adding phosphate-buffered saline. After this process, the concentration of pro-inflammation cytokines was detected in each sample. To detect the pro-inflammation cytokines concentration in cells, the culture medium was collected.

### Measurement of neutrophil and monocyte in BAL fluids

After anesthesia and semi-excision of the trachea, plastic cannulas were inserted and lung tissues were washed with 2 ml of 0.9% NaCl to obtain bronchoalveolar lavage (BAL) samples. These operations were repeated three times. Cells in the BAL fluids were attached to the glass slides cytospin preparation. Neutrophils and monocytes were obtained using the Differential Quick Stain kit (Jiancheng, Nanjing, China) and counted by optical microscopy.

### Determination of VDR mRNA

Total RNA was extracted with TRIZOL reagent (Takara, Dalian, China) according to the manufacturer's instruction. RNA concentration was tested by spectrometric analysis. VDR and β-actin were examined by Real Time PCR following the manufacturer's instructions (Takara Perfect Real Time). Amplification and detection were carried out by using Bio-Rad My iQ detection system (Edinburgh Biological Science and Technology Development co.). Relative quantification of target cDNA was determined by arbitrarily setting the control value to 1 and changes in cDNA content of a sample were expressed as a multiple thereof. Genes and primers are listed as follows: The sequences of the rat VDR primers were 5′-CACAGGCTTCCACTTCAATGCTA-3′ (forward) and 5′-TCATGCCGATGTCCACACAG-3′ (reverse). The sequences of the human VDR primers were 5′-TCAATCAAACGCTATGCCTCTC-3′ (forward) and 5′-CCAGCCCCTCACAACTCAA-3′ (reverse). The sequences of the rat β-actin primers were 5′-ACGGTCAGGTCATCACTATCGG-3′ (forward) and 5′-GCACTGTGTTGGCATAGAGGTC-3′ (reverse). The sequences of the human β-actin primers were 5′- TGGCACCCAGCACAATGAA-3′ (forward) and 5′-CTAAGTCATAGTCCGCCTAGAAGCA-3′ (reverse).

### A549 cell culture and treatment

A human lung epithelial cell line, A549 (obtained from ATCC, Rockville, MD, USA), was maintained in RPMI 1640 medium supplemented with 100 U/ml of penicillin-streptomycin and 10% fetal bovine serum (FBS) at 37°C in a humidified atmosphere containing 5% CO_2_ and 95% air. After incubated in the presence or absence of calcitriol (10^−10^ M, 10^−8^ M, 10^−6^ M) and dexamethasone (10^−6^ M) 48 h, seawater (0.25 ml per 1 ml total volume) were added to A549 cells and the cells were stimulated for indicated time.

### RPMVEC isolation, treatment and identification

Isolation and culture of RPMVEC were performed according to the previous methods with some modification [19], [20]. Briefly, the fresh lung was aseptically removed from the killed rats and washed. After the pleura and the outer edges of the lung lobe were cut off, the specimens of tissue (1.5 mm^3^) obtained from the lung surface were carefully plated into tissue culture dishes containing DMEM supplemented with 20% FBS, 100 U/ml of penicillin-streptomycin and 25 µg/ml of endothelial cell growth supplement (Upstate Biotechnology Inc, Lake Placid, NY, USA) at 37°C in a humidified atmosphere containing 5% CO_2_ and 95% air. Sixty hours later, the residue lung tissues were removed. After that, the medium was changed every 2 days. When monolayer cells were achieved, the cells were passaged with a 0.25% solution of trypsin. Experimental data were obtained from cells between passage 2 and 3. After incubated in the presence or absence of calcitriol (10^−6^ M) and dexamethasone (10^−6^ M) 48 h, seawater (0.25 ml per 1 ml total volume) were added to cells and the cells were stimulated for indicated time.

Primary RPMVEC were identified according to their characteristic morphology such as staining with anti-CD31 antibody.

### RhoA activation assay

Activation of RhoA proteins was determined as described in previous studies [21], [22]. This pull-down assay (Upstate Biotechnology Inc, Lake Placid, NY, USA) uses the RhoA binding domain (RBD) from the effector protein Rhotekin as a probe to isolate the active forms of RhoA. Briefly, extracted lungs were homogenized in the lysis buffer (50 mM Tris, pH 7.2, 1% TritonX-100, 0.5% sodium deoxycholate, 0.1% SDS, 500 mM NaCl, 10 mM MgCl_2_, protease inhibitor cocktail). Lysates were then clarified by centrifugation at 16300×g for 5 min in a centrifuge. Supernatants were then immediately removed and added to the aliquots of Rhotekin-RBD beads. Samples were incubated on a rotator for 1 h, washed with washing buffer, and analyzed by SDS-Page and Western blotting with RhoA antibody.

RhoA activity of cells was determined as follows. Cells were cultured in 100 mm culture dishes. After stimulation, the cells were lysed in lysis buffer (25 mM HEPES, pH 7.5, 150 mM NaCl, 1% Igepal CA-630, 10 mM MgCl_2_, 1 mM EDTA, 2% glycerol, 2 µg/ml each of leupeptin and aprotinin, 1 µl/ml phenylmethylsulphonylfluoride and 0.5 µl/ml DTT). The cellular proteins were harvested by scraping with a rubberpoliceman. The lysates were centrifuged at 16300×g for 5 min.The protein extracts (25 µg of each) were incubated while rotating at 4°C for 3 h with an equal volume of Rhotekin-RBD bound to glutathione-agarose beads. The beads were washed three times with washing buffer and activated RhoA bound to the beads or total RhoA in cell extracts was detected using western blot analysis with RhoA antibody.

### Western blot analysis

In brief, the lysates extracted from the harvested lung tissues or cultured cells were removed by centrifugation at 14800×g for 20 min at 4°C. Protein concentration was determined by BCA protein assay kit. Protein was boiled in loading buffer, resolved in 10% SDS-polyacrylamide gels, electrotransferred to nitrocellulose membranes, and blocked with 5% non-fat milk in TBST. The membrane was incubated overnight with the indicated primary antibody β-actin (1∶5,000 dilution), NF-κB p65 (1∶1,000 dilution), pNF-κB p65 (1∶1,000 dilution), RhoA (1∶1,000 dilution), MYPT1 (1∶1,000 dilution) and pMYPT1 (1∶1,000 dilution). The secondary antibody (goat anti-mouse or anti-rabbit IgG, 1∶5,000) was incubated and the relative content of target protein was detected by the enhanced chemiluminescent (ECL) detection system (Amersham Pharmacia Biotech, Arlington Heights, IL, USA) according to the manufacturer's protocol.

### Measurement of A549 and RPMVEC cell monolayer permeability

The permeability measurement was performed as previously described [20], [23]. A549 or RPMVEC (2×10^5^ cells/well) were cultured on collagen coated Transwell insert to contrast an in vitro model of a cell monolayer. The insert were placed into 24-well plates containing 500 µl medium. After the cell monolayer formation and at the end of stimulation, 100 µl FITC-dextran (2,000-kDa, Sigma) was added into the insert and incubated for 1 h. The insert was then removed and 100 µl medium collected from the bottom chamber. The fluorescent density of samples was analyzed by a fluorospctrophotometer (LS-50B, PE, USA).

### Statistical analysis

All data are expressed as mean ±SD. Statistical comparisons were made using Mann-Whitney U-test or one-way ANOVA followed by Dunnett's test. Differences were considered statistically significant at *P*<0.05.

## Results

### Effects of seawater stimulation on VDR expression in rat lung, A549 cells and RPMVECs

Stimulation with seawater in rat lung, A549 cells and RPMVECs significantly up-regulates VDR mRNA (*P*<0.001versus normal group) expression, as detected by real-time RT-PCR (Figure S1).

### Effects of calcitriol on seawater aspiration-induced lung injury, edema and vascular leakage

**Figure 1:**
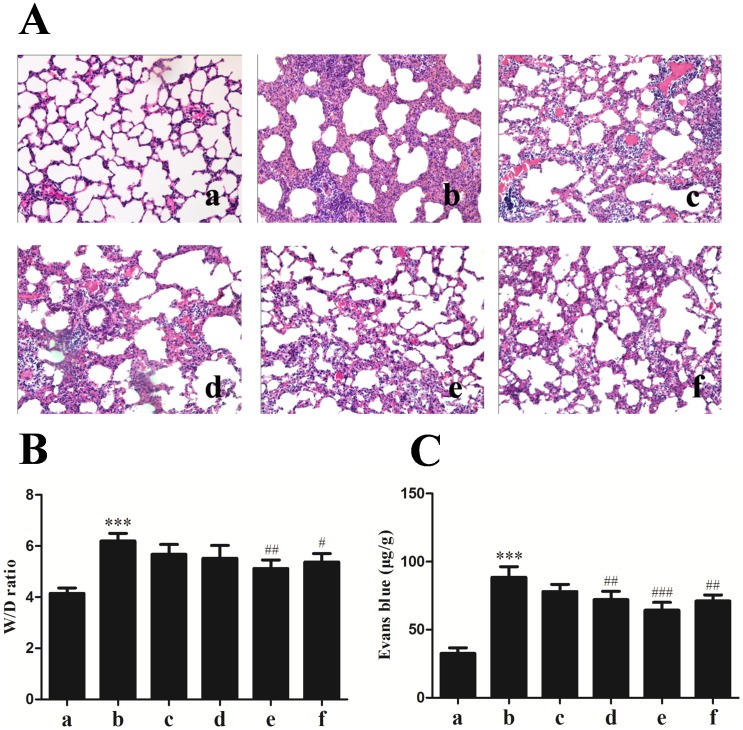
Effects of calcitriol on seawater aspiration-induced lung histopathologic changes (A), wet/dry weight ratios (B) and Evans blue leakage (C). Histopathologic, W/D ratio and Evans blue leakage examination of the lung tissues were performed at 4 h after seawater aspiration. (a) normal group; (b) seawater group; (c)–(e) 1 µg/kg, 5 µg/kg and 25 µg/kg calcitriol groups; (f) dexamethasone group. n = 8, ***P<0.001 versus group a, # P<0.05, ## P<0.01, ### P<0.001 versus group b. Histopathologic changes showed pulmonary edema, infiltration of inflammatory cells, hemorrhage and alveolar distortion in seawater group. In calcitriol group, lung injury was significantly alleviated compared with seawater group and dose dependence was observed. Dexamethasone also alleviated lung damage compared with seawater group, but no significant difference compared with 25 µg/kg calcitriol group. (Hematoxylin-eosin stain, 20×).

In order to investigate whether calcitriol could change the lung histopathology, we tested the effect of peroral administration of calcitriol at 1, 5 and 25 µg/kg respectively. (Figure 1A). Compared with the control, seawater instillation for 4 h induced pulmonary edema, infiltration of inflammatory cells in lung tissues and alveoli, as well as alveolar thickening, distortion and hemorrhage. In contrast, administration of calcitriol could significantly improve the lung injury. The histological changes in different dose group and dexamethasone group have a different degree miniature, but there is no significant difference between calcitriol group and dexamethasone group.

To further evaluate the effect of calcitriol on seawater aspiration-induced lung edema, we measured the lung wet/dry weight ratios of each group (Figure 1B). Compared with normal group, the W/D ratios significantly increased in seawater aspiration group (*P*<0.001). Although the effect of calcitriol on lung edema alleviation was dose dependence, only the 25 µg/kg calcitriol group has significant difference versus seawater aspiration group (*P*<0.01). In addition, there is no significant difference between dexamethasone group and seawater aspiration group (*P*>0.05).

To assess the severity of the lung vascular leakage, we observed the leak index of Evans blue (Figure 1C). Compared with normal group, the Evans blue leakage significantly increased in seawater aspiration group (*P*<0.001). The Evans blue leakage reduced in calcitriol group by dose dependence manner and the effects of dexamethasone were similar to that of calcitriol.

### Effects of calcitriol on neutrophil and monocyte recruitment in the lung

There are many types of cells in the BAL fluids such as inflammatory cells, erythrocytes and epithelial cells. Considering that accumulation of inflammatory cells is a hallmark of inflammation in seawater aspiration-induced ALI, we further investigated inflammatory cell differentiation in BAL and assessed the effect of calcitriol on inflammatory cells (Figure S2). As shown in the figure, calcitriol inhibited dose-dependently neutrophil recruitment compared with seawater group. However, calcitriol had no significant effects on monocyte recruitment in lung tissue (*P*>0.05).

### Effects of calcitriol on the expression of pro-inflammation cytokines and NF-κB p65 activation in rat lung after seawater administration

**Figure 2:**
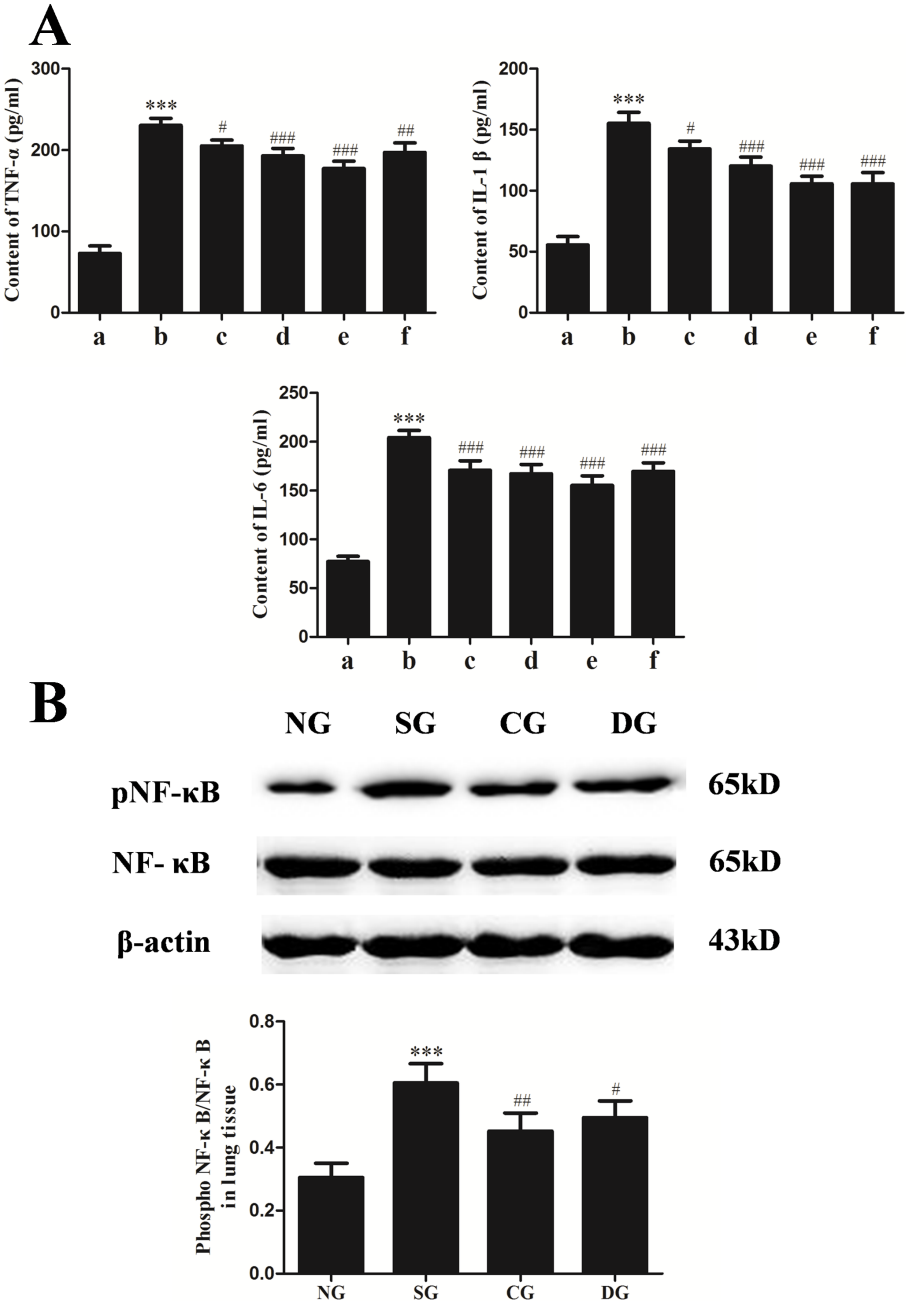
Effects of calcitriol on the levels of TNF-α, IL-1β and IL-6 (A) in lung tissues. All data were obtained at 4 h after seawater aspiration. (a) normal group; (b) seawater group; (c)–(e) 1 µg/kg, 5 µg/kg and 25 µg/kg calcitriol groups; (f) dexamethasone group. n = 8, ***P<0.001 versus group a, # P<0.05, ## P<0.01, ### P<0.001 versus group b. Effects of calcitriol on the NF-κB p65 phosphorylation (B) in lung tissues. Proteins were obtained at 4 h after seawater aspiration. The ratios of pNF-κB versus NF-κB were obtained for each group to examine the pNF-κB content. NG: normal group; SG: seawater group; CG: 25 µg/kg calcitriol group; DG: dexamethasone group. n = 6, ***P<0.001 versus group NG, # P<0.05, ## P<0.01 versus group SG.

We assessed the effects of calcitriol on the levels of TNF-α, IL-1β and IL-6 induced by seawater inflammation in the lung tissue (Figure 2A). As shown in the figure, TNF-α, IL-1β and IL-6 contents markedly increased after seawater stimulation. Compared with the seawater group, calcitriol significantly inhibited the expression of these pro-inflammation cytokines and it was clear that the expression of these pro-inflammation cytokines decreased as the calcitriol dosage increased.

NF-κB p65 plays an important role in the inflammatory responses. To identify the activation of the NF-κB pathway in the lung tissues, NF-κB p65 activity was measured by western blot. As shown in Figure 2B, the activation of NF-κB p65 in seawater group was significantly increased compared with normal group (*P*<0.001), and calcitriol obviously inhibited the phosphorylation of NF-κB p65 after seawater administration (*P*<0.01 versus seawater group). However, no markedly difference between calcitriol group and dexamethasone group were observed (*P*>0.05).

### Effects of calcitriol on RhoA activation and Rho kinase activity in rat lung stimulated by seawater

**Figure 3:**
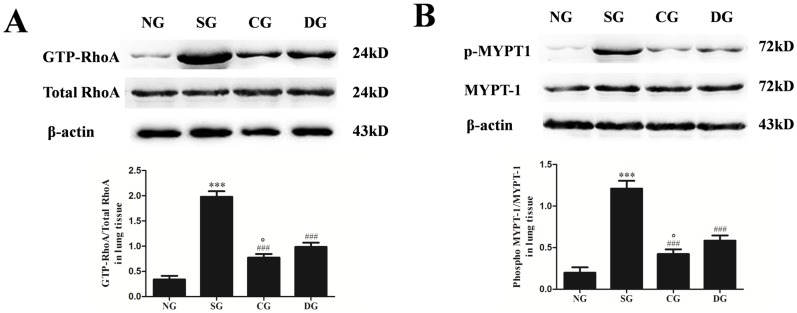
Effects of calcitriol on the GTP-RhoA and p-MYPT1 in lung tissues. Proteins were obtained at 4 h after seawater aspiration. The ratios of GTP-RhoA versus total-RhoA and p-MYPT1 versus MYPT1 were obtained for each group to examine the GTP-RhoA (A) and p-MYPT1 (B) contents. NG: normal group; SG: seawater group; CG: 25 µg/kg calcitriol group; DG: dexamethasone group. n = 6, ***P<0.001 versus group NG, ### P<0.001 versus group SG, ° P<0.05 versus group DG.

To evaluate the activation of the RhoA-Rho kinase pathway in the lung tissues, RhoA and myosin phosphatase target subunit 1 (MYPT-1) activities were measured by western blot (Figure 3). MYPT-1 is the regulatory subunit of myosin light chain phosphatase which works as a downstream RhoA effector [24]. So we measured ROCK activity by evaluating phosphorylation of MYPT-1. As expected, GTP-RhoA and MYPT-1 phosphorylation were enhanced by seawater treatment (*P*<0.001 versus normal group). Calcitriol treatment significantly attenuated the increase of GTP-RhoA and p-MYPT1 after seawater administration (*P*<0.001 versus seawater group). Moreover, the treatment effect of calcitriol was better than dexamethasone (*P*<0.05).

### Effects of calcitriol on the expression of pro-inflammation cytokines in A549 cells stimulated by seawater

As shown in Figure S3, seawater stimulation enhances the basal production of TNF-α, IL-1β and IL-6. Compared with seawater group, calcitriol inhibited dose-dependently the expression of these pro-inflammation cytokines.

### Effects of calcitriol on NF-κB p65 activation and RhoA/ROCK pathway in A549 cells after seawater treatment

**Figure 4:**
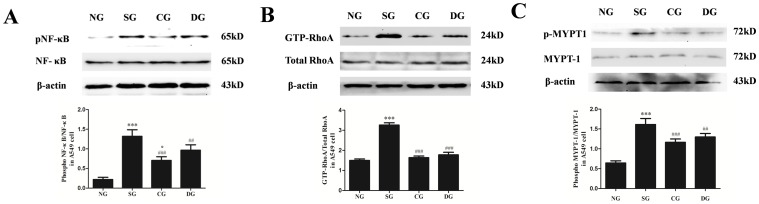
Effects of calcitriol on NF-κB p65 activation and RhoA/ROCK pathway in A549 cells. After 4 h seawater treatment, proteins were obtained for western blot. The ratios of pNF-κB versus NF-κB, GTP-RhoA versus total-RhoA and p-MYPT1 versus MYPT1 were obtained for each group to examine the pNF-κB, GTP-RhoA and p-MYPT1 content. NG: normal group; SG: seawater group; CG: 10^−6^ M calcitriol group; DG: dexamethasone group. n = 6, ***P<0.001 versus group NG, ## P<0.01, ### P<0.001 versus group SG, ° P<0.05 versus group DG.

To assess the capacity of calcitriol to inhibit activation of NF-κB p65 in A549 cells, NF-κB p65 activity was measured by western blot (Figure 4A). Compared with normal group, phosphorylation of NF-κB p65 in seawater group was significantly enhanced (*P*<0.001). Calcitriol treatment significantly attenuated the increase of pNF-κB p65 after seawater administration (*P*<0.001 versus seawater group). In addition, this effect of calcitriol was more significant than dexamethasone (*P*<0.05).

To identify the activation of the RhoA/ROCK pathway in A549 cells stimulated by seawater, GTP-RhoA (Figure 4B) and p-MYPT1 (Figure 4C) activities were measured by western blot. As shown in the figure, seawater administration enhanced the increasing expression of GTP-RhoA and p-MYPT1 (*P*<0.001 versus normal group). Both calcitriol and dexamethasone significantly inhibited RhoA activation and MYPT-1 phosphorylation after seawater stimulation. However, no markedly difference between calcitriol group and dexamethasone group were observed (*P*>0.05).

### RPMVEC characteristics

The primary cells were verified as endothelial cells by the capillary-like structure and typical cobble-stone appearance at confluence (Figure S4A). Sub-cultured cells were in accordance with endothelial cells in morphology. Moreover, the sub-cultured cells displayed closely packed, homogeneous, short spindle or polygonal in shape (Figure S4B). Meanwhile, our isolated cells were confirmed as endothelial cells by positive expression of CD31 (Figure S4C).

### Effects of calcitriol on RhoA/ROCK pathway in RPMVECs stimulated by seawater

**Figure 5:**
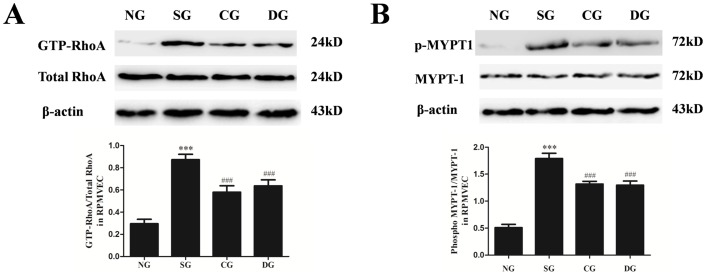
Effects of calcitriol on RhoA/ROCK pathway in RPMVECs. Proteins were obtained at 4 h after seawater administration. The ratios of GTP-RhoA versus total-RhoA and p-MYPT1 versus MYPT1 were obtained for each group to examine the GTP-RhoA (A) and p-MYPT1 (B) contents. NG: normal group; SG: seawater group; CG: 10^−6^ M calcitriol group; DG: dexamethasone group. n = 6, ***P<0.001 versus group NG, ### P<0.001 versus group SG.

The lung tissue barrier consists of epithelial cells and endothelial cells, so we also examined the activation of the RhoA/ROCK pathway in RPMVECs stimulated by seawater. GTP-RhoA (Figure 5A) and p-MYPT1 (Figure 5B) activities were measured by western blot. Compared with the normal group, seawater significantly induced the increase of GTP-RhoA and p-MYPT1 (*P*<0.001). Calcitriol and dexamethasone evidently inhibited RhoA activation and MYPT-1 phosphorylation (*P*<0.001 versus seawater group), but no markedly difference between calcitriol group and dexamethasone group were observed (*P*>0.05).

### Effects of calcitriol on monolayer permeability in A549 cells and RPMVECs stimulated by seawater

**Figure 6:**
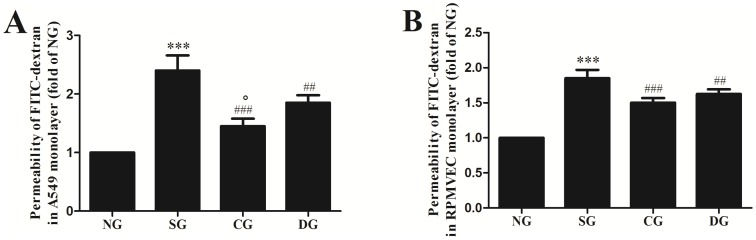
Effects of calcitriol on monolayer permeability in A549 cells and RPMVECs. A549 cells and RPMVECs were treated for 40 min with seawater in the presence or absence of calcitriol and dexamethasone. After A549 (A) and RPMVEC (B) monolayer formation, cells were treated for 40 min with seawater. The permeability was shown as fold of NG. NG: normal group; SG: seawater group; CG: 10^−6^ M calcitriol group; DG: dexamethasone group. n = 6, ***P<0.001 versus group NG, ## P<0.01, ### P<0.001 versus group SG, ° P<0.05 versus group DG.

Cell contraction is a RhoA/ROCK-dependent event. To investigate the effect of calcitriol on monolayer permeability of A549 cells (Figure 6A) and RPMVECs (Figure 6B), we examined the FITC-dextran flux across the monolayer. Application of seawater to A549 cells and RPMVECs resulted in significant increase in FITC-dextran flux (*P*<0.001 versus normal group). Prior treatment with calcitriol and dexamethasone both attenuated the increase of FITC-dextran flux but calcitriol has stronger effect than dexamethasone in A549 cells (*P*<0.05). However, no significant difference between calcitriol group and dexamethasone group were observed in RPMVECs (*P*>0.05).

## Discussion

In this study, we investigated the role of calcitriol for seawater aspiration-induced ALI. The results showed that seawater stimulation led to lung histopathologic changes, lung edema, vascular leakage, inflammatory cells recruitment and release of pro-inflammation cytokines. Moreover, seawater administration induced the expression of VDR, activated NF-κB and RhoA/ROCK pathways both in lung tissues and cells. Meanwhile, calcitriol treatment significantly inhibited the seawater-induced ALI.

Although there are several pharmacological studies for patients with ALI and ARDS, these treatments did not significantly reduce mortality [25]. Calcitriol, the active metabolite of vitamin D, bind to the vitamin D-receptor and subsequently to responsive genes [26]. It has many pharmacological properties. A lot of studies confirmed that calcitriol suppresses inflammatory responses in several cell types such as human blood cells, dendritic cell, human dermal fibroblasts and human nasal polyp fibroblasts [27], [28], . In addition, it has been shown that calcitriol inhibits neutrophil recruitment in LPS-induced ALI [17].

Seawater aspiration-induced ALI belongs to direct ALI. Our previous studies showed that the hyperosmolar seawater that touched directly to the lung epithelial cells induced release of pro-inflammation cytokines and infiltration of inflammation cells [31], [32]. Moreover, seawater also significantly induced pulmonary epithelial-endothelial barrier injury and the increase of lung tissue permeability as evidenced by bronchoalveolar lavage fluid protein, W/D ratio and the leak index of Evans blue and some of the damage index were most obvious after 4–6 hours seawater exposure [33], [34], [35]. These studies explained the mechanism of seawater aspiration-induced ALI to a certain extent.

NF-κB pathway plays an important role in the inflammation responses and the activation of this pathway is also a crucial part in seawater aspiration-induced ALI [32]. After the seawater stimulation, many released cytokins may lead to the degradation of IkBs, and this can induce NF-κB phosphorylation and translocation from the cytoplasm to the nucleus in which they bind with target genes and regulate their transcription [36]. This process results in the significant increase of NF-κB-dependent pro-inflammation cytokines, and these cytokines eventually promote lung tissue barrier permeability and enhance inflammatory cells recruitment to the area of injury. There are many upstream signaling molecules involved in NF-κB activation. Previous studies suggest that activation of NF-κB by RhoA/ROCK pathway indicates a potential role for this pathway in inflammation responses [37], [38], [39]. So we chose RhoA/ROCK pathway as a potential therapeutic target to inhibit inflammation response in seawater-induced ALI. RhoA belongs to monomeric GTP-binding proteins of the Rho family and it works through translocation to the cell plasma membrane. In our present study, we demonstrated that pretreatment of calcitriol inhibited NF-κB phosphorylation and reduced the release of related pro-inflammatory cytokines. Meanwhile, administration of calcitriol also inhibited RhoA activation. Furthermore, the effect of calcitriol was better than already known therapeutic drug dexamethasone.

Another critical issue of seawater aspiration-induced ALI is pulmonary edema. Previous studies confirmed that pulmonary edema was resulted from the pulmonary epithelial-endothelial barrier injury and the barrier permeability increase [40]. In the models of LPS and ventilator-induced lung injury, the activation of Rho pathway leads to increase of lung tissue barrier permeability. In addition, activation of Rho pathway plays a critical part in the formation of stress fiber and paracellular gap in LPS-stimulated endothelial cells [41], [42], [43]. Moreover, many other studies demonstrated that RhoA and its downstream effect or ROCK can regulate many essential cellular functions including cell contraction and modulation of actin cytoskeleton [44]. Therefore, we hypothesized that RhoA/ROCK pathway is critical in the pulmonary epithelial-endothelial barrier permeability increase. We found that seawater stimulation induced the increase of W/D ratio and the leak index of Evans blue in lung tissues and led to monolayer permeability in A549 cells and RPMVECs. At the same time, the RhoA/ROCK pathway was activated. Accordingly, this result confirmed that the activation of RhoA/ROCK pathway take part in the increase of lung tissue barrier permeability. However, pretreatment of calcitriol reduced the W/D ratio and the leak index of Evans blue after seawater aspiration. Meanwhile, calcitriol also inhibited RhoA activation and reduced monolayer permeability both in epithelial cells and endothelial cells.

## Conclusions

Our data demonstrated therapeutic effects of calcitriol in seawater aspiration-induced ALI. Calcitriol affected the two main characteristics of seawater aspiration-induced ALI: inflammation and pulmonary epithelial-endothelial barrier injury. The effects of calcitriol to attenuate ALI induced by seawater were through inhibiting activation of NF- κB and RhoA/ROCK pathways. It may be considered as a potential agent in the prevention and therapy of the seawater aspiration-induced ALI.

## Supporting Information

Figure S1 **Quantification of VDR mRNA expression by real-time RT-PCR in rat lung (A), A549 cell (B) and RPMVEC (C) untreated or treated for 4 h with seawater.** mRNA levels are shown as arbitrary units normalized to β-actin expression. n = 8, ***P<0.001 versus NG. NG: normal group. SG: seawater group.
(TIF)

Figure S2 **Effects of calcitriol on neutrophil and monocyte recruitment in the lung.** All data were obtained at 4 h after seawater stimulation. (A) seawater group; (B)–(D) 1 µg/kg, 5 µg/kg and 25 µg/kg calcitriol groups; (E) dexamethasone group. n = 8, *P<0.05, **P<0.01 versus group A.
(TIF)

Figure S3 **Effects of calcitriol on the levels of TNF-α, IL-1β and IL-6 in A549 cells.** All data were obtained at 4 h after seawater stimulation. (a) normal group; (b) seawater group; (c) – (e) 10^−10^M, 10^−8^M and10^−6^M calcitriol groups; (f) dexamethasone group. n = 8, ***P<0.001 versus group a, # P<0.05, ## P<0.01, ### P<0.001 versus group b.
(TIF)

Figure S4 **Primary RPMVEC (A) and sub-cultured cells (B) were observed by an inverted microscope (magnification 10×).** C: RPMVEC were identified by the expression of CD31 (magnification 20×).
(TIF)
