# Supplementary figures and images for: 1α,25-Dihydroxyvitamin D3 Ameliorates Seawater Aspiration-Induced Acute Lung Injury via NF-κB and RhoA/Rho Kinase Pathways
Source: PLoS One. 2014 Aug 13;9(8):e104507. doi: 10.1371/journal.pone.0104507 (PMC4132109; doi:10.1371/journal.pone.0104507)

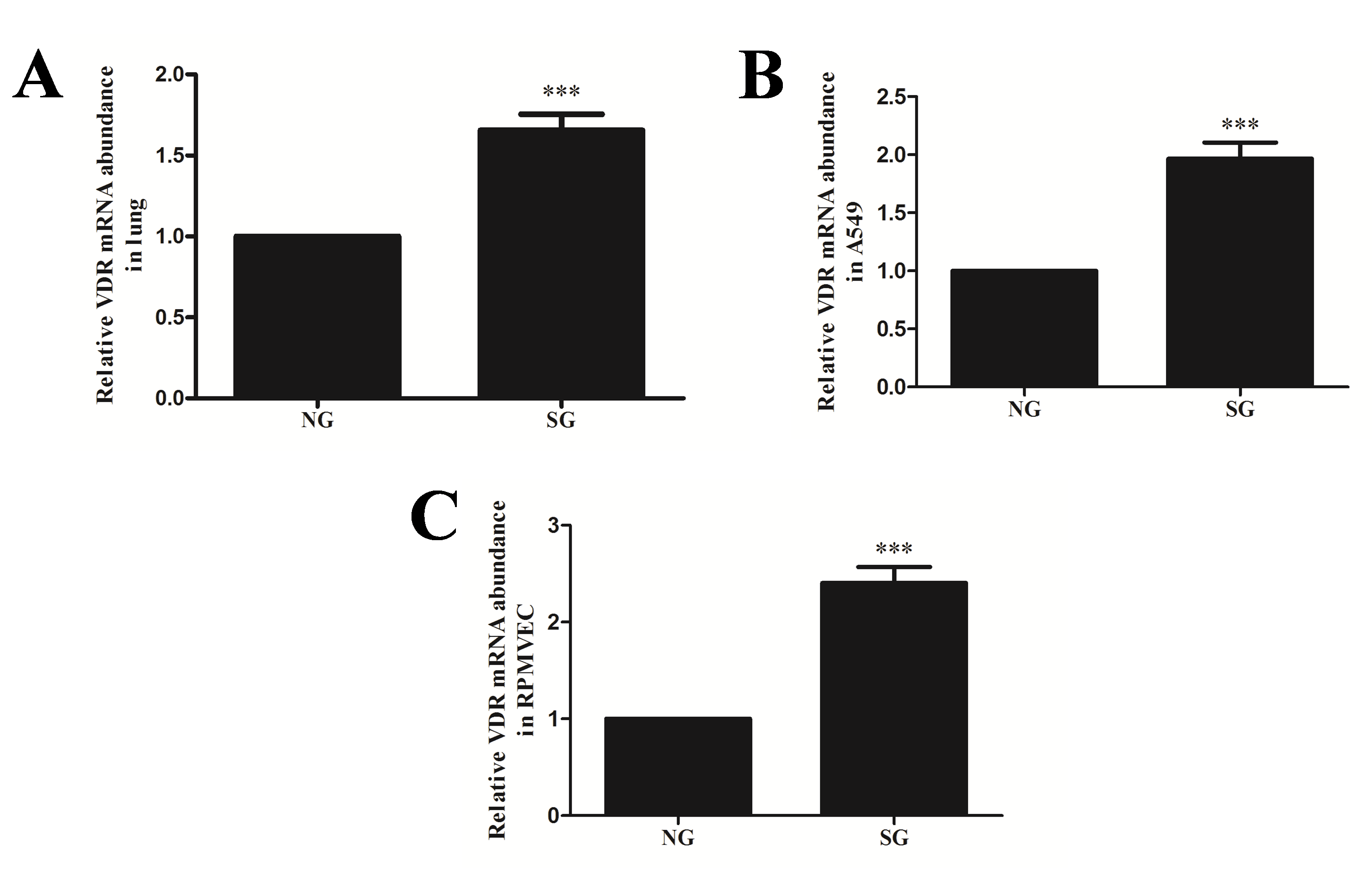

Supplement: Figure S1 — Quantification of VDR mRNA expression by real-time RT-PCR in rat lung (A), A549 cell (B) and RPMVEC (C) untreated or treated for 4 h with seawater. mRNA levels are shown as arbitrary units normalized to β-actin expression. n = 8, ***P<0.001 versus NG. NG: normal group. SG: seawater group. (TIF) [file pone.0104507.s001.tif]

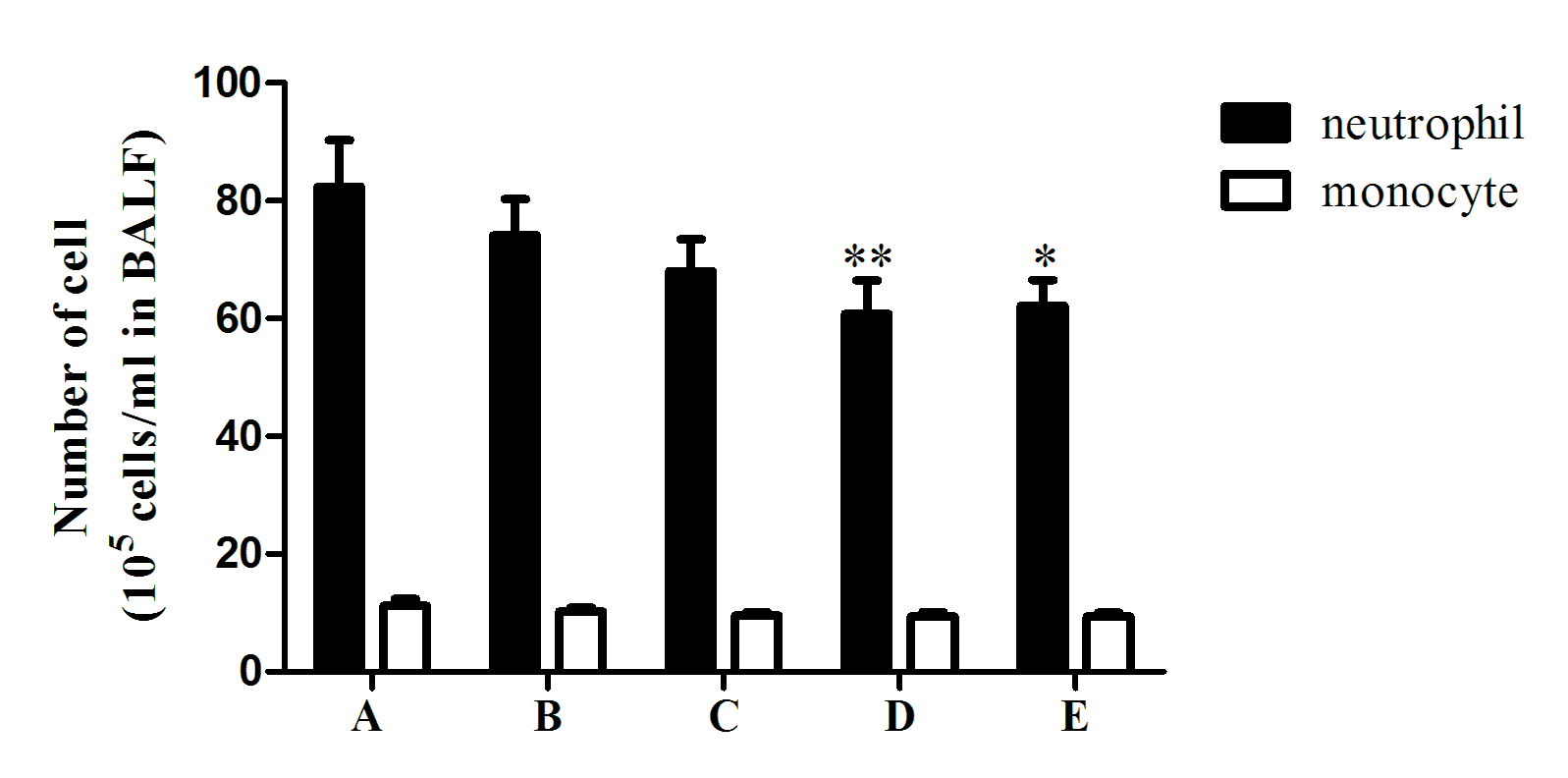

Supplement: Figure S2 — Effects of calcitriol on neutrophil and monocyte recruitment in the lung. All data were obtained at 4 h after seawater stimulation. (A) seawater group; (B)–(D) 1 µg/kg, 5 µg/kg and 25 µg/kg calcitriol groups; (E) dexamethasone group. n = 8, *P<0.05, **P<0.01 versus group A. (TIF) [file pone.0104507.s002.tif]

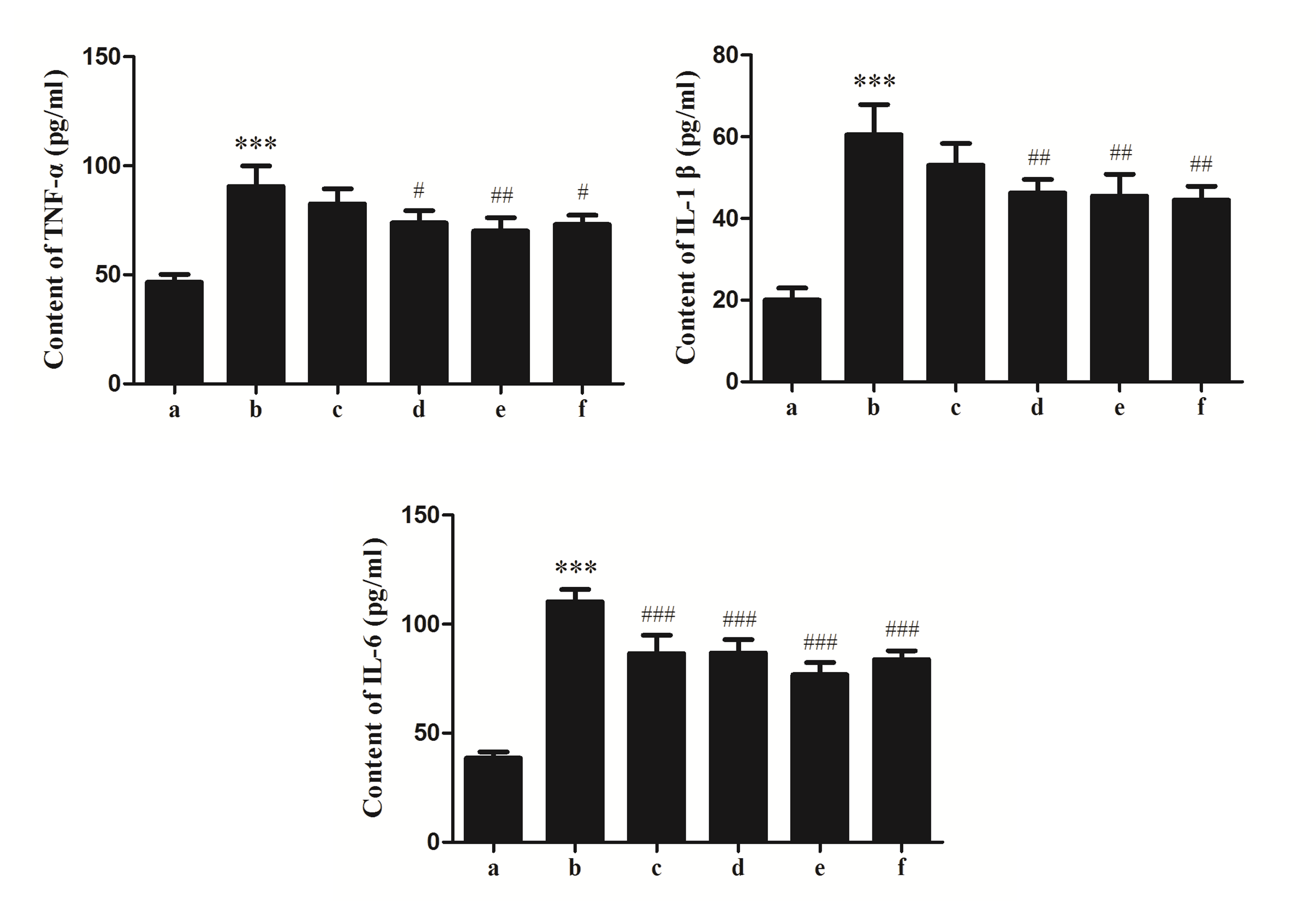

Supplement: Figure S3 — Effects of calcitriol on the levels of TNF-α, IL-1β and IL-6 in A549 cells. All data were obtained at 4 h after seawater stimulation. (a) normal group; (b) seawater group; (c) – (e) 10−10M, 10−8M and10−6M calcitriol groups; (f) dexamethasone group. n = 8, ***P<0.001 versus group a, # P<0.05, ## P<0.01, ### P<0.001 versus group b. (TIF) [file pone.0104507.s003.tif]

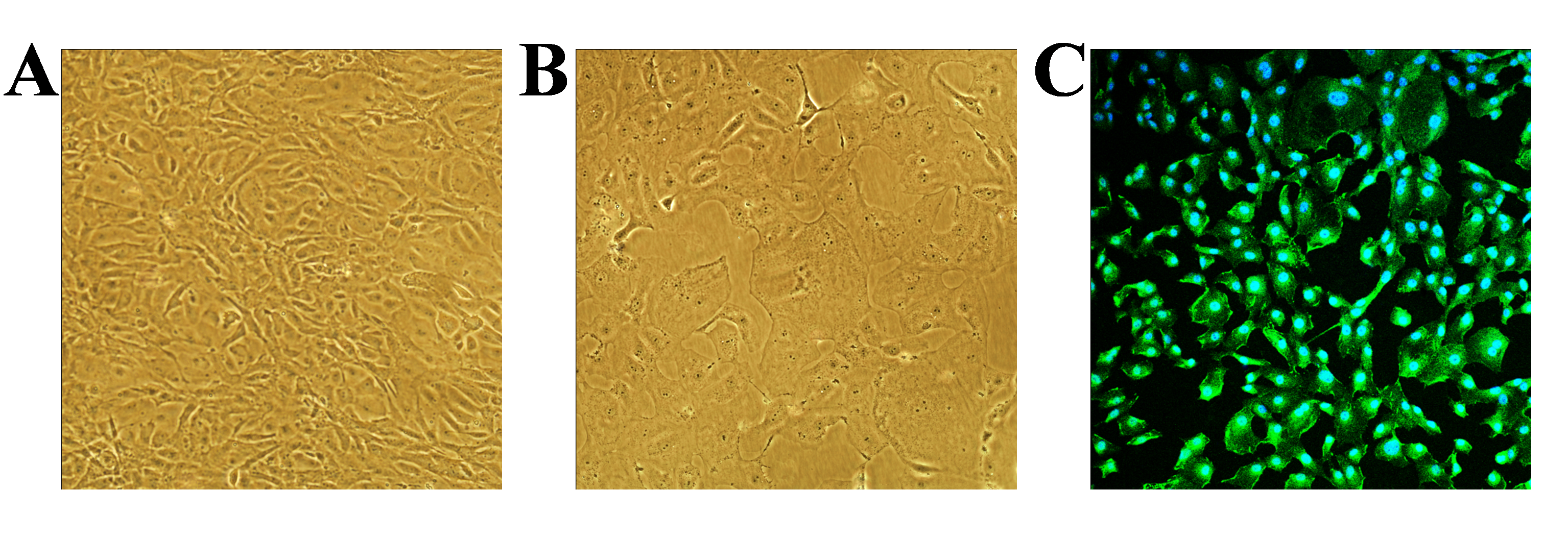

Supplement: Figure S4 — Primary RPMVEC (A) and sub-cultured cells (B) were observed by an inverted microscope (magnification 10×). C: RPMVEC were identified by the expression of CD31 (magnification 20×). (TIF) [file pone.0104507.s004.tif]
